# Snoring might be a warning sign for metabolic syndrome in nonobese Korean women

**DOI:** 10.1038/s41598-023-44348-4

**Published:** 2023-10-09

**Authors:** Suk Won Chang, Ha Young Lee, Hyun Seung Choi, Jung Hyun Chang, Gil Chai Lim, Ju Wan Kang

**Affiliations:** 1 Department of Otorhinolaryngology, Jeju National University College of Medicine, Jeju, Korea; 2 Department of Otorhinolaryngology, National Health Insurance Service Ilsan Hospital, 100 Ilsan-ro, Ilsandong-gu, Goyang, 10444 Korea; 3 Department of Otorhinolaryngology, Yongin Severence Hospital, Yonsei University College of Medicine, 363, Dongbaekjukjeon-daero, Giheung-gu, Yongin, 16995 Korea

**Keywords:** Metabolic syndrome, Comorbidities, Sleep disorders

## Abstract

Metabolic syndrome (MetS) is an underlying cause of various diseases and is strongly associated with mortality. In particular, it has been steadily increasing along with changes in diet and lifestyle habits. The close relationship between sleep apnea and MetS is well established. In addition, these two diseases share a common factor of obesity and have a high prevalence among obese individuals. Nevertheless, the association can vary depending on factors, such as race and sex, and research on the relatively low obesity rates among East Asians is lacking. This study aimed to investigate the association between snoring and MetS in nonobese Koreans. A total of 2478 participants (827 men and 1651 women) were enrolled in the Korean National Health and Nutrition Examination Survey from 2019 to 2020. We used the National Cholesterol Education Program Adult Treatment Panel III criteria for MetS and a snoring questionnaire. Logistic regression analysis was used to measure the association between MetS and various confounding factors according to age and sex in participants with body mass index (BMI) < 23 kg/m^2^. MetS was significantly higher in participants with snoring than in those without snoring (26.9% vs. 19.6%; *p* = 0.007). In multivariate logistic regression analysis, age (odds ratio [OR] 1.070, 95% confidence interval [CI] 1.059–1.082, *p* < .001), sex (OR 1.531, 95% CI 1.139–2.058, *p* = 0.005), and snoring (OR 1.442, 95% CI 1.050–1.979, *p* = 0.024) were significantly associated with MetS in patients with a BMI < 23 kg/m^2^. Finally, regression analysis showed that snoring was significantly associated with MetS in women with a BMI of less than 23 kg/m^2^, especially with younger ages (40–49 years, OR 4.449, 95% CI 1.088 to 18.197, *p* = 0.038). Snoring was closely associated with MetS in women aged 40–50 years with a BMI of less than 23 kg/m^2^ compared to other participants. However, the association was not found in women aged 60 and over. Therefore, sufficient consideration should be given to the possibility of MetS when snoring is present in nonobese middle-aged Asian women.

## Introduction

Metabolic syndrome (MetS) is a condition characterized by a series of metabolic abnormalities, with at least three abnormal metabolic health indicators, including blood sugar, blood pressure, serum lipids, and body fat distribution. MetS is closely related not only to diseases, such as obesity, hypertension, diabetes, and hyperlipidemia, but also to mortality, and it is known to occur when risk factors that can cause such diseases accumulate^1–3^. It is known that the prevalence of MetS worldwide ranges from 20 to 25%, and it is increasing not only in developed countries, but also in developing countries^4^. In addition, there is growing concern about the prevalence of MetS not only in adults, but also in children and adolescents^5^.

Obesity is closely related to MetS and contributes significantly to its development^6^. Furthermore, obesity is one of the most important risk factors for obstructive sleep apnea (OSA)^7^. Therefore, OSA has been reported to be highly correlated with various health problems, as well as a high association with MetS^8,9^. Polysomnography is the gold standard for the diagnosis of sleep apnea. However, owing to the cost and inconvenience of the test, it is difficult for everyone to undergo the examination. As a result, many patients with sleep apnea are undiagnosed, and various screening tools are being proposed to address this issue. Snoring is the most commonly observed symptom in patients with OSA and is present in 70–95% of the patients with OSA^10–12^. Conversely, 20–70% of the patients with snoring are known to have OSA^13^. Therefore, some studies have shown a significant correlation between snoring, an important indicator of OSA, and MetS^14,15^. However, these correlations can vary depending on various demographic factors, such as age, sex, and race^16^. Therefore, the fact that existing studies have been conducted primarily in Western populations with higher levels of obesity compared to East Asians suggests a need for a further study.

This study aimed to investigate the association between snoring and MetS among Koreans aged 40 years and above with a BMI of less than 23 kg/m^2^ who demonstrated normal weight, using data from the Korean National Health and Nutrition Examination Survey (KNHANES) from 2019 to 2020.

## Materials and methods

### Study population

**Figure 1:**
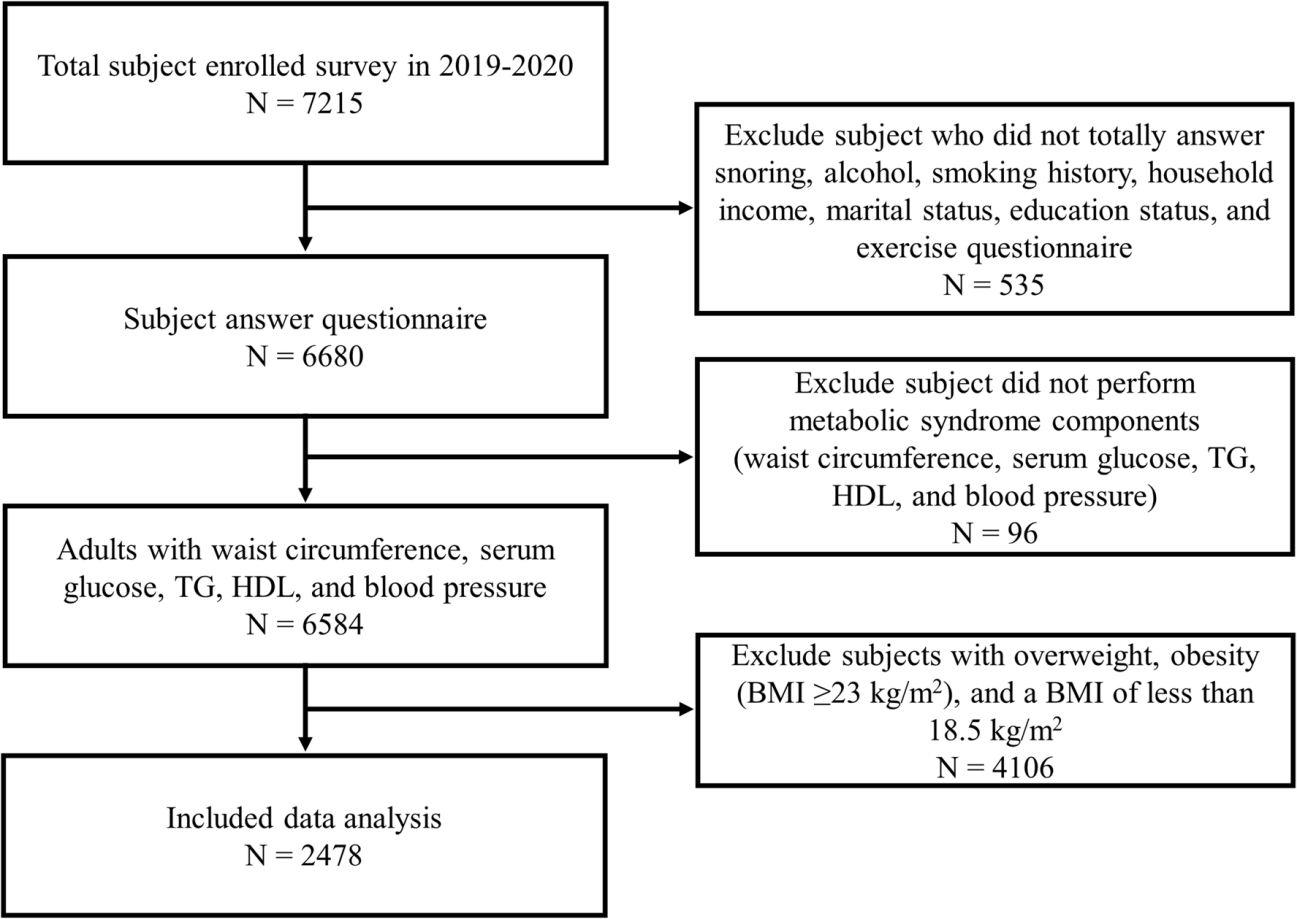
Flowchart of study participants. Among the 7215 participants who participated in the Korea National Health and Nutrition Examination Survey from 2019 to 2020, 6680 participants answered snoring, and other clinical variables questionnaire were included. Finally, a total of 2478 participants, excluding those who met the exclusion criteria, were analyzed in this study.

We conducted an analysis of 2478 participants (827 men and 1651 women) corresponding to the inclusion criteria of 7215 adults aged 40 years or older who responded to snoring surveys in the 2019 and 2020 KNHANES data. Detailed information about this study was provided to subjects or legal guardians and informed consent was obtained from all enrolled subjects. The exclusion criteria were as follows: of the 7215 participants, 6680 were included, excluding 535 who did not respond to the alcohol, smoking history, household income, marital status, education status, and exercise questionnaires. Among them, 6584 patients were enrolled, excluding 96 who did not undergo tests for MetS (waist circumference, serum glucose, triglycerides [TG], high-density lipoprotein [HDL], and blood pressure). A total of 2478 patients were enrolled and analyzed, excluding 4106 patients with a BMI of 23 kg/m^2^ or higher (Fig. 1). All methods and protection of personal information were performed in accordance with the Declaration of Helsinki. This study was approved by the Jeju National University Institutional Review Board.

### Measurements of variables

We conducted a questionnaire survey on the participants’ sex, age, smoking history, alcohol history, monthly household income, education, marital status, and exercise habits. Additionally, a questionnaire was administered to determine whether the participants experienced snoring. In the questionnaire, subjects were asked to select either “Yes” or “No” to the following question: “Is your snoring louder than the sound of a conversation or loud enough to be heard in the next room?” We used the National Cholesterol Education Program Adult Treatment Panel III criteria to define MetS^17^:

- Obesity (waist circumference > 102 cm [men], > 88 cm [women])
- Hyperglycemia (fasting plasma glucose ≥ 100 mg/dL) or pharmacologic treatment
- Dyslipidemia (TG ≥ 150 mg/dL) or pharmacologic treatment
- HDL < 40 mg/dL (men), < 50 mg/dL (women) or pharmacologic treatment
- Hypertension (systolic blood pressure > 130 mmHg or > 85 mmHg diastolic) or pharmacological treatment

Subjects were classified as having MetS when they satisfied at least three of the five criteria listed above. We also defined the standard for subjects with normal weight as having a BMI between 18.5 and 22.9 kg/m^2^^18^, and conducted the analysis using subjects with normal weight. As for exercise habit, if the exercise habit was moderate or higher (exercise habit was defined as participation in more than 30 min of moderate physical activity twice a week for over a year), it was classified as yes, and less exercise was classified as no.

### Statistical analysis

We used t-test and chi-square test to compare the two groups according to the presence or absence of snoring. For participants with BMI of less than 23 kg/m^2^, the association between snoring and MetS according to sex and age was analyzed using multivariate logistic regression. The Statistical Package for the Social Sciences statistical software package version 17 (SPSS Inc., Chicago, IL, USA) was used for statistical analysis. In all analyses, the *p*-value was considered two-tailed, and the statistical significance was set at *p* < 0.05.

## Results

A total of 2478 participants (827 men and 1651 women) were included in this study. Men had a higher prevalence of snoring than women (16.8% vs. 7.8%; *p* < 0.001). BMI was significantly higher in the group with snoring than in the group without snoring (21.4 ± 1.3 vs. 20.9 ± 1.5; *p* < 0.001), and the TG level was also significantly higher in the group with snoring (126.1 ± 100.5 vs. 109.9 ± 73.9; *p* = 0.011). HDL level was lower in the group with snoring (53.2 ± 13.3 vs 56.0 ± 13.3; *p* = 0.001), and systolic (123.2 ± 18.0 vs. 120.2 ± 18.5; *p* = 0.010) and diastolic pressures (76.1 ± 10.0 vs. 74.2 ± 9.8; *p* = 0.003) were higher in the snoring group (Table 1).

**Table 1:** Prevalence of snoring and related demographic and clinical variables in Korean adults.

	Snoring		*p* value
	Absent (N = 2210)	Present (N = 268)	
Sex^#^			< 0.001
Male	688 (31.1%)	139 (51.9%)	
Female	1522 (68.9%)	129 (48.1%)	
Age (years)*	58.7 ± 12.0	59.8 ± 11.0	0.150
BMI (kg/m^2^)*	20.9 ± 1.5	21.4 ± 1.3	< 0.001
HTN^#^	482 (21.8%)	67 (25.0%)	0.267
DM^#^	207 (9.4%)	31 (11.6%)	0.296
Smoking^#^	446 (20.2%)	81 (30.2%)	< 0.001
Alcohol^#^	970 (43.9%)	143 (53.4%)	< 0.001
Waist circumference (cm)*	76.6 ± 6.4	79.8 ± 5.7	< 0.001
Fasting glucose (mg/dL)*	99.1 ± 20.0	103.6 ± 26.7	0.007
TG (mg/dL)*	109.9 ± 73.9	126.1 ± 100.5	0.011
HDL cholesterol (mg/dL)*	56.0 ± 13.3	53.2 ± 13.3	0.001
Systolic pressure (mmHg)*	120.2 ± 18.5	123.2 ± 18.0	0.010
Diastolic pressure (mmHg)*	74.2 ± 9.8	76.1 ± 10.0	0.003
Number of metabolic syndrome components^#^			0.021
0	675 (30.5%)	60 (22.4%)	
1	639 (28.9%)	73 (27.2%)	
2	462 (20.9%)	63 (23.5%)	
3	263 (11.9%)	47 (17.5%)	
4	165 (7.5%)	24 (9.0%)	
5	6 (0.3%)	1 (0.4%)	
Total calorie intake (kcal)*	1708.9 ± 717.4	1819.3 ± 765.7	0.018
Fat intake (g)*	39.0 ± 28.6	38.4 ± 26.5	0.744
Exercise habit^#^			0.009
No	1915 (86.7%)	216 (80.6%)	
Yes	295 (13.3%)	52 (19.4%)	
Marital status^#^			0.005
Married	2110 (95.5%)	266 (99.3%)	
Single	100 (4.5%)	2 (0.7%)	

BMI, body mass index; HTN, hypertension; DM, diabetes mellitus; TG, triglyceride; HDL, high-density lipoprotein.

^#^Categorical variables are expressed as number (%).

*Continuous variables are represented as mean ± standard deviation.

**Figure 2:**
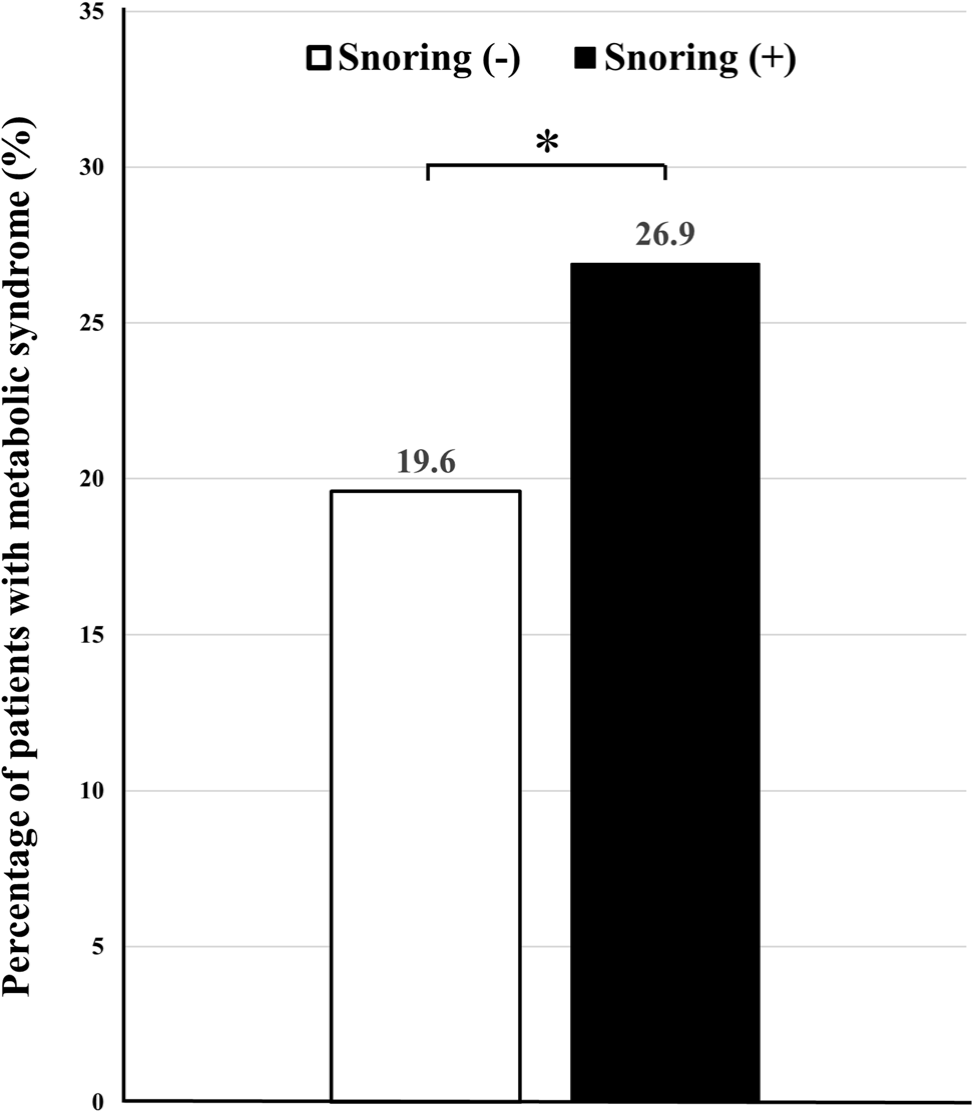
Percentage of participants with metabolic syndrome according to the presence or absence of snoring among participants with body mass index < 23 kg/m^2^.

Chi-square test was performed to investigate whether the frequency of MetS differed depending on the presence of snoring. The results showed that even in the normal-weight group with a BMI of less than 23 kg/m^2^, the number of MetS patients was significantly higher in the group with snoring than in the group without snoring (26.9% vs. 19.6%, respectively, *p* = 0.007) (Fig. 2). Multivariate logistic regression analysis was conducted to analyze the association between each variable and MetS. The results showed that MetS significantly increased with older age, women, higher BMI, higher calorie intake, lower fat intake, and snoring (Table 2).

**Table 2:** Multivariate logistic regression analysis of associated factors of the metabolic syndrome in patients with BMI < 23 (N = 2478).

Variables	Number of subjects with Mets	OR	95% CI	*p*
Age		***1.070***	***1.059–1.082***	** < *0.001***
Sex (women)	1651	***1.531***	***1.139–2.058***	***0.005***
BMI		***1.339***	***1.246–1.452***	** < *0.001***
Smoking (+)	527	1.151	0.845–1.568	0.372
Alcohol (+)	1113	0.835	0.657–1.061	0.140
Exercise habit (no)	347	0.714	0.499–1.023	0.066
Marital status (unmarried)	102	0.735	0.339–1.596	0.437
Total calorie intake		***1.000***	***1.000–1.001***	***0.023***
Fat intake		***0.988***	***0.982–0.995***	** < *0.001***
Snoring (+)	268	***1.442***	***1.050–1.979***	***0.024***

OR, odds ratio; CI, confidence interval.

Bold italics indicates statistical significance (*p* < 0.05).

**Figure 3:**
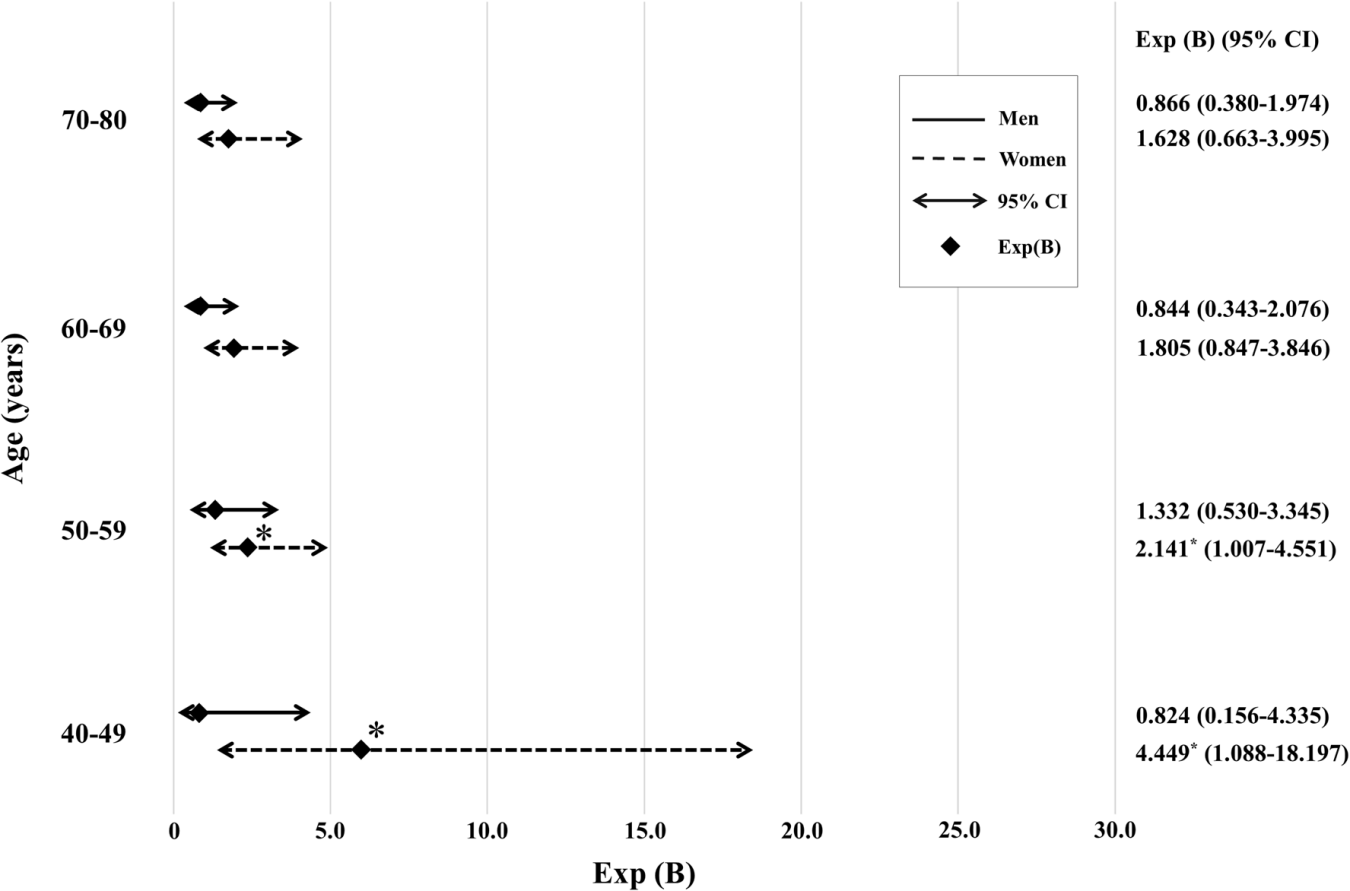
Multivariate logistic regression analysis of associated factors of the metabolic syndrome according to age group in patient with body mass index < 23 kg/m^2^. OR, odds ratio. Bold italics indicates statistical significance (*p* < 0.05).

To minimize the impact of sex and age, the participants were classified into different groups according to age and sex, and a multivariate analysis was conducted again (Table 3). Despite focusing on the normal-weight group with a BMI of less than 23 kg/m^2^, the study still demonstrated a statistically significant relationship between BMI and MetS. Despite the statistically significant association observed between snoring and MetS in the entire study population, no significant association was found between snoring and MetS in men across all age groups. However, there was a significant association between snoring and MetS in certain age groups of women, despite the lack of a significant association in men across all age groups. Specifically, significant results were found in women aged 40–49 years and 50–59 years, with a stronger association observed in the younger age groups (Table 3, Fig. 3).

**Table 3:** Multivariate logistic regression analysis of associated factors of the metabolic syndrome according to age group in patients with BMI < 23 (N = 2478).

	70–80 years				60–69 years				50–59 years				40–49 years			
	**Men**		**Women**		**Men**		**Women**		**Men**		**Women**		**Men**		**Women**	
Variables	**OR**	***p***	**OR**	***p***	**OR**	***p***	**OR**	***p***	**OR**	***p***	**OR**	***p***	**OR**	***p***	**OR**	***p***
Number of subjects	**264**		**292**		**217**		**370**		**184**		**466**		**162**		**523**	
Age	1.024	0.590	***1.086***	***0.022***	0.995	0.931	***1.097***	***0.031***	1.005	0.946	1.093	0.068	1.158	0.244	0.925	0.455
BMI	***1.455***	***0.001***	1.087	0.281	***1.722***	***0.001***	***1.296***	***0.008***	1.157	0.297	***1.385***	***0.006***	1.422	0.248	***1.831***	***0.025***
Smoking	0.840	0.583	0.681	0.616	1.692	0.144	0.619	0.389	1.447	0.355	1.064	0.918	0.946	0.934	1.954	0.416
Subject with Mets (N)	26		3		32		5		25		4		6		2	
Alcohol	1.790	0.066	0.814	0.589	0.648	0.219	***0.447***	***0.009***	1.696	0.251	0.806	0.457	1.254	0.791	0.403	0.136
Subject with Mets (N)	46		14		31		18		31		26		9		5	
Exercise habit	0.799	0.673	0.710	0.497	0.851	0.778	1.016	0.970	0.725	0.539	0.629	0.270	0.578	0.530	0.000	0.996
Subject with Mets (N)	6		7		5		10		6		8		2		0	
Marital status	0.000	1.000	1.601	0.704	0.000	0.999	1.288	0.845	0.459	0.323	0.649	0.689	1.355	0.726	0.000	0.998
Subject with Mets (N)	0		2		0		1		2		1		2		0	
Total calorie intake	0.999	0.087	1.001	0.089	1.000	0.655	1.000	0.390	1.000	0.528	1.001	0.054	1.000	0.839	0.999	0.145
Fat intake	1.014	0.154	0.982	0.069	0.990	0.275	***0.982***	***0.024***	0.982	0.072	***0.978***	***0.012***	1.002	0.892	1.019	0.313
Snoring	0.866	0.732	1.628	0.288	0.844	0.713	1.805	0.126	1.332	0.542	***2.141***	***0.048***	0.824	0.819	***4.449***	***0.038***
Subject with Mets (N)	10		13		8		15		9		12		2		3	

OR, odds ratio; N, number; Mets, Metabolic syndrome.

Bold italics indicates statistical significance (*p* < 0.05).

## Discussion

This study confirmed the significant association between snoring and MetS. In particular, even in nonobese individuals with a BMI of less than 23 kg/m^2^, snoring showed a significant association with MetS. In addition, in the analysis by sex, the association between snoring and MetS could not be confirmed in nonobese men with a BMI of less than 23 kg/m^2^. However, in nonobese women, the younger the age, the higher the association confirmed, especially in women in their 40 s.

Several previous studies have reported an association between sleep apnea and MetS, but the precise mechanism has yet to be identified, and several possible explanations exist. The first possible mechanism is that intermittent hypoxia increases the sympathetic nervous system and affects MetS development^19^. This is similar to how chronic insomnia increases cortisol levels, leading to sympathetic nervous system activation^20^. Another possible explanation is that vascular inflammation caused by sleep apnea affects MetS development through an inflammatory response^21^. Similarly, transient ischemic tissue releases free radicals, and systematic inflammation and cytokine release by oxidative stress may contribute to the development of MetS^22^.

Obesity is strongly correlated with MetS and is a well-known risk factor for OSA^23^. Li et al.^24^ reported an interaction effect between obesity, snoring, and MetS. A similar study reported that snoring is a risk factor for MetS, and that this association weakens when obesity-related indicators, such as BMI, are considered confounders^25^. As such, there seems to be a complex association between obesity, snoring, and MetS, and a study has shown that these associations also exist in nonobese people. Kim et al.^26^ reported that even in the group with a BMI of less than 25 kg/m^2^ in Korea, the incidence of MetS increased when there was snoring in both men and women, although the odds ratio was lower than that in the group with a BMI of 25 kg/m^2^ or more. In a meta-analysis on the association between snoring and MetS, there was a significant association between snoring and MetS in both men and women^2^. However, in our study, this tendency was confirmed only in women, and there was a difference in that there was no tendency in men. A previous study has shown different correlations depending on sex, which is similar to our results. Lee et al*.* compared glycosylated hemoglobin (HbA1c) levels between people with and without snoring and found that in women, HbA1c levels were higher in those with snoring than in those without snoring. However, no significant differences were observed between men and women^27^. Therefore, further research is required to determine the relationship between snoring and MetS.

In this study, a significant relationship between snoring and MetS was observed in women. There are several possible explanations for the sex-related differences. Jordan et al.^28^ explained that this was due to the physiological differences between the anatomy of the upper airway and movement of the pharynx according to sex. Another explanation is that female sex hormones play a preventive role in sleep apnea, resulting in the differences between men and women. Therefore, in postmenopausal women, the risk of sleep apnea increases due to a lack of female hormones compared to that in premenopausal women^29^. However, further studies are required to elucidate the exact physiological mechanisms.

As shown in the results of this study, there was a previous study on the association between MetS and sleep apnea in lean Asian adults. Goto et al.^30^ reported a positive association between snoring frequency and the prevalence of hypertension in subjects with a BMI of less than 22.8 kg/m^2^. In contrast, our study results did not confirm a significant association between snoring and hypertension, one of the MetS components, in subjects with a BMI of less than 23 kg/m^2^. The reason for this is that although the subjects were of the same Asian race, there was a possibility that the subjects did not precisely match. For instance, in the study by Goto et al.^30^, the subjects were middle-aged adults, while the age of our study subjects ranged from 40 to 80 years; therefore, there may have been differences in the study results. There was also a difference in the method of analyzing the results. Goto et al.^30^ analyzed the association between systolic/diastolic blood pressure and snoring frequency. In contrast, our study analyzed the association between snoring without distinguishing between snoring frequency and hypertension/high blood pressure.

Another study reported a positive association between nocturnal intermittent hypoxia, a surrogate marker for obstructive sleep apnea, and MetS in non-obese Asian adults with a BMI of under 25 kg/m^2^^31^. In this study, the age group of the subjects was not consistent with our study, and the standard for non-overweight adults was a BMI of less than 25 kg/m^2^, which was also different from ours. Muraki et al.^31^ reported that there was an association between nocturnal intermittent hypoxia, high blood pressure, high triglyceride level, and the presence of two or more metabolic risk factors. However, multivariate logistic regression analysis on the association between MetS components (hyperglycemia, dyslipidemia, and low high-density lipoprotein) and snoring showed that only low HDL was significantly associated with snoring in women aged 40–49 years in our study (Supplemental Table 1). Therefore, additional research is needed to confirm the association between snoring and obstructive sleep apnea with MetS.

The advantages of this study were that the number of participants was relatively large, and the relationship between snoring and MetS was analyzed in nonobese participants by classifying the degree of obesity based on BMI. The strength of our analysis was the ability to minimize the effects of age and sex by conducting subgroup analyses. By examining the relationships between each variable and MetS within specific age and sex groups, we were able to identify more specific patterns and reduce the potential confounding effects of age and sex on our results. Our study confirmed that the relationship between snoring and MetS was significantly strong in Korean women with a normal BMI.

However, this study had several limitations. First, since our study was cross-sectional, it was difficult to conclude a causal relationship between snoring and MetS. Therefore, additional prospective studies are required to analyze the causal relationship between snoring and MetS. Second, in the evaluation of snoring, an objective evaluation, such as polysomnography, was not performed using a self-report questionnaire, and simple snoring patients without OSA were not distinguished. However, the self-report questionnaire currently used in this study is known to be a reliable measure for predicting sleep apnea in other epidemiological studies and could be used instead of polysomnography^32^. Finally, this study confirmed that the relationship between snoring and MetS was strong in women. However, the number of cases that responded as having the snoring defined by us was extremely low in younger women. This lower frequency may have amplified the statistical significance. Further research may be needed to address this matter. Moreover, there is still a lack of clear mechanistic explanation. Also, the authors focused on individuals with a BMI of less than 23 kg/m^2^ in this study. Therefore, when compared to the entire population, some differences were observed in demographic characteristics (Supplemental Table 2). It is believed that examining the impact of these demographic differences or conducting comparisons between the two groups in the future could be meaningful.

## Conclusion

This study confirmed the association between snoring and MetS in Korean women aged ≥ 40 years who were not obese. However, the association was not found in woman aged 60 years and over. Therefore, even for nonobese women, regular snoring should be considered a possibility of MetS, and its evaluation is necessary.

### Supplementary Information

Supplementary Table 1.

Supplementary Table 2.

## Data Availability

All available data generated or analyzed during this study are included in this published article. Other raw data are not available because of regulation of data sharing in the Republic of Korea.
