# Supplementary material for: Snoring might be a warning sign for metabolic syndrome in nonobese Korean women
Source: Sci Rep. 2023 Oct 9;13:17041. doi: 10.1038/s41598-023-44348-4 (PMC10562394; doi:10.1038/s41598-023-44348-4)
Supplement: Supplementary file 1 — Supplementary Table 1. [file 41598_2023_44348_MOESM1_ESM.docx]

Supplemental table1. Multivariate logistic regression analysis between snoring and metabolic syndrome components according to age group in patient with BMI<23 (N=2478)

|  | 70-80 years | | | | 60-69 years | | | | 50-59 years | | | | 40-49 years | | | |
| --- | --- | --- | --- | --- | --- | --- | --- | --- | --- | --- | --- | --- | --- | --- | --- | --- |
|  | **Men** | | **Woman** | | **Men** | | **Woman** | | **Men** | | **Woman** | | **Men** | | **Woman** | |
| MetS components | **OR** | ***p*** | **OR** | ***p*** | **OR** | ***p*** | **OR** | ***p*** | **OR** | ***p*** | **OR** | ***p*** | **OR** | ***p*** | **OR** | ***p*** |
| Hyperglycemia | 1.082 | 0.833 | 0.825 | 0.684 | 1.199 | 0.643 | 1.145 | 0.728 | 1.574 | 0.282 | 1.304 | 0.466 | 0.890 | 0.798 | 1.165 | 0.762 |
| Dyslipidemia | 0.671 | 0.402 | 0.637 | 0.425 | 0.364 | 0.055 | 1.612 | 0.319 | 0.392 | 0.103 | 1.006 | 0.990 | 2.143 | 0.108 | 1.247 | 0.684 |
| Low HDL | 1.354 | 0.491 | 1.289 | 0.655 | 1.688 | 0.329 | 1.085 | 0.869 | 2.819 | 0.067 | 1.074 | 0.872 | 0.754 | 0.639 | ***2.875*** | ***0.008*** |
| Hypertension | 1.138 | 0.734 | 1.976 | 0.270 | 1.289 | 0.516 | 0.967 | 0.930 | 1.700 | 0.198 | 1.319 | 0.435 | 0.648 | 0.389 | 1.335 | 0.564 |

Abbreviations: OR, odds ratio.

Bold italics indicates statistical significance (*p*<.05).

Adjusted for age, BMI, smoking, alcohol consumption, and exercise habit
