# Supplementary material for: Snoring might be a warning sign for metabolic syndrome in nonobese Korean women
Source: Sci Rep. 2023 Oct 9;13:17041. doi: 10.1038/s41598-023-44348-4 (PMC10562394; doi:10.1038/s41598-023-44348-4)
Supplement: Supplementary file 2 — Supplementary Table 2. [file 41598_2023_44348_MOESM2_ESM.docx]

Supplemental table 2. demographic characteristics between study participant and population

|  | Study participants  (N=2478) | All population  (N=7215) | *p* value |
| --- | --- | --- | --- |
| Sex^#^ |  |  | < 0.001 |
| Male | 827 (33.4%) | 3068 (42.5%) |  |
| Female | 1651 (66.6%) | 4147 (57.5%) |  |
| Age(years) ^*^ | 58.8 ± 11.9 | 59.5 ± 11.6 | 0.011 |
| BMI(kg/m^2^)^*^ | 21.0 ± 1.5 | 24.2 ± 3.4 | < 0.001 |
| HTN^#^ | 549 (22.2%) | 2378 (33.0%) | < 0.001 |
| DM^#^ | 238 ( 9.6%) | 994 (13.8%) | < 0.001 |
| Smoking^#^ | 527 (21.3%) | 1776 (24.6%) | 0.001 |
| Alcohol^#^ | 1113 (44.9%) | 3413 (47.4%) | 0.037 |
| Waist Circumference (cm) ^*^ | 77.0 ± 6.4 | 85.4 ± 9.7 | < 0.001 |
| Fasting Glucose (mg/dL)^*^ | 99.5 ± 20.9 | 104.7 ± 24.7 | < 0.001 |
| TG (mg/dL) ^*^ | 111.7 ± 77.4 | 136.2 ± 101.6 | < 0.001 |
| HDL cholesterol (mg/dL) ^*^ | 55.7 ± 13.3 | 51.6 ± 12.6 | < 0.001 |
| Systolic Pressure (mmHg) ^*^ | 120.5 ± 18.5 | 123.9 ± 17.4 | < 0.001 |
| Diastolic Pressure (mmHg) ^*^ | 74.4 ± 9.8 | 76.5 ± 10.1 | < 0.001 |
| Snoring^#^ | 268 (10.8%) | 1356 (18.8%) | < 0.001 |
| Number of metabolic syndrome components^#^ |  |  | < 0.001 |
| - 0 | 735 (29.7%) | 1228 (17.0%) |  |
| - 1 | 712 (28.7%) | 1589 (22.0%) |  |
| - 2 | 525 (21.2%) | 1538 (21.3%) |  |
| - 3 | 310 (12.5%) | 1396 (19.3%) |  |
| - 4 | 189 ( 7.6%) | 1063 (14.7%) |  |
| - 5 | 7 ( 0.3%) | 401 ( 5.6%) |  |
| Total Calorie Intake (kcal) ^*^ | 1720.8 ± 723.4 | 1766.3 ± 767.9 | 0.009 |
| Fat intake (g) ^*^ | 38.9 ± 28.4 | 38.8 ± 29.2 | 0.867 |
| Exercise habit^#^ |  | | 0.577 |
| - No | 347 (14.0%) | 1045 (14.5%) |  |
| - Yes | 347 (14.0%) | 1045 (14.5%) |  |
| Marital status^#^ |  | | 0.529 |
| - Married | 2376 (95.9%) | 6948 (96.3%) |  |
| - Single | 102 ( 4.1%) | 266 ( 3.7%) |  |

Abberviation: BMI: body mass index; HTN: hypertension; DM: diabetes mellitus; TG: triglyceride; HDL; high density lipoprotein

^#^Categorical variables are expressed as number(%).

^*^Continuous variables are represented as mean ± standard deviation
